# Rapid, Single-Molecule Assays in Nano/Micro-Fluidic Chips with Arrays of Closely Spaced Parallel Channels Fabricated by Femtosecond Laser Machining

**DOI:** 10.3390/s140815400

**Published:** 2014-08-20

**Authors:** Brian K. Canfield, Jason K. King, William N. Robinson, William H. Hofmeister, Lloyd M. Davis

**Affiliations:** Center for Laser Applications, University of Tennessee Space Institute, 411 B. H. Goethert Parkway, MS 35, Tullahoma, TN 37388, USA; E-Mails: jason.king@nist.gov (J.K.K.); quantum.penguin@gmail.com (W.N.R.); whofmeis@utsi.edu (W.H.H.); ldavis@utsi.edu (L.M.D.)

**Keywords:** high-throughput, microfluidic, rapid readout, fluorescence correlation spectroscopy, femtosecond laser machining

## Abstract

Cost-effective pharmaceutical drug discovery depends on increasing assay throughput while reducing reagent needs. To this end, we are developing an ultrasensitive, fluorescence-based platform that incorporates a nano/micro-fluidic chip with an array of closely spaced channels for parallelized optical readout of single-molecule assays. Here we describe the use of direct femtosecond laser machining to fabricate several hundred closely spaced channels on the surfaces of fused silica substrates. The channels are sealed by bonding to a microscope cover slip spin-coated with a thin film of poly(dimethylsiloxane). Single-molecule detection experiments are conducted using a custom-built, wide-field microscope. The array of channels is epi-illuminated by a line-generating red diode laser, resulting in a line focus just a few microns thick across a 500 micron field of view. A dilute aqueous solution of fluorescently labeled biomolecules is loaded into the device and fluorescence is detected with an electron-multiplying CCD camera, allowing acquisition rates up to 7 kHz for each microchannel. Matched digital filtering based on experimental parameters is used to perform an initial, rapid assessment of detected fluorescence. More detailed analysis is obtained through fluorescence correlation spectroscopy. Simulated fluorescence data is shown to agree well with experimental values.

## 1. Introduction

The necessity of rapidly screening the interactions of biomolecules of interest with extensive libraries of small molecules as part of the pharmaceutical drug discovery pipeline has grown tremendously. Vast libraries of drug candidates may be produced through combinatorial chemical synthesis techniques, but reagent costs remain the major limiting factor and drive miniaturization of the assay platform. Furthermore, the inventory of therapeutic targets has ballooned owing to increased comprehension of the genetic causes underlying diseases. Thus, many techniques regarding the ability to perform assays on large numbers of viable candidates quickly and cheaply, collectively referred to as high-throughput screening, have been developed [1,2].

Protein analysis methods abound with parallel microfluidic schemes. Gel electrophoresis has been used in several large-dimension polymer microfluidic chips to separate proteins [3,4], and in a 96-well titer plate format [5]. Rare cells were screened in a device consisting of 384 hemispherical microchannels in glass [6]. A zone-plate array was integrated with 64 parallel microchannels, forming a novel architecture, and fluorescence from drops of dye in the microchannels was detected at the rate of 184,000 drops per second [7]. Fluorescence-based detection of 1 million drops over 7 min in a digital droplet-based reactor configuration with wide-field imaging has been employed to study polymerase chain reactions [8]. The use of a fast, sensitive single-photon avalanche diode array in a high-throughput detection technique has been reported recently [9].

High-throughput systems are not restricted to microscale dimensions. Nanofluidic polymer devices are also appearing in the literature. One scheme employs a fiber-pulling technique to reduce a bundle of microscale capillaries to nanoscale dimensions [10]. Another inventive method involves forming nanochannels on a Petri dish lid and casting them in a polymer film [11]. A different approach uses photolithography and oxygen plasma to etch channels with submicron depths in thermoplastic polymers [12]. However, the development of a highly parallel fluidic platform, whether microscale or nanoscale, that integrates the ability to probe all channels simultaneously with single-molecule sensitivity remains an active research field.

To this end, a system was sought to link two titer plates through a polymer fluidic interconnect comprised of highly parallel microchannels [13], forming the background to this paper. Further research on this method had aimed to shrink the microchannel cross-sectional dimensions to ∼1 μm^2^ and increase the density to 192 channels, so that the interconnect could be used in conjunction with standard 96-well titer plates. However, attempts to make polymer microfluidic devices with imprint lithography encountered difficulties. For microchannel widths less than 5 μm, either the polymer material stuck within the molds, destroying channel separations, or the microchannels became highly deformed during bonding [13].

Because of these difficulties, polymer microfluidic devices with the desired dimensions were unavailable. We have instead used direct writing by an amplified femtosecond laser to make highly parallel prototype devices for single-molecule fluorescence measurements. The advantages of fs lasers for machining, which include localized damage and avoidance of heating, are well known [14]. Structural changes in the material result from nonlinear absorption of the pulse energy and only occur where the intensity exceeds a threshold value. Hence, it is possible to create features smaller than the diffraction limit [15,16]. Depending on the pulse energy, the expanding, transient plasma created around the beam focus may force material into the surrounding volume, thereby increasing the density, or explosively expel it if near the surface. *In situ* altered material may then be removed through selective chemical etching by solutions, such as hydrofluoric acid or hot, concentrated potassium hydroxide [17].

In this paper we discuss the fabrication of prototype microfluidic devices containing arrays of >150 microchannels ∼1 μm wide and separated by 2 μm, although in principle, finer nanoscale channels are achievable. A completed microfluidic device is mounted in a custom wide-field microscope with a line-generating diode laser source. An electron-multiplying CCD (EM-CCD) camera serves as the fluorescence detector. A matched digital filter is applied to the collected data as a rapid, initial assessment of fluorescence signals. Fluorescence autocorrelation for individual channels, which is computationally more intense, may be performed once fluorescence signals have been verified. A simulated fluorescence data set is generated and analyzed according to the same conditions and found to agree substantially with experiment.

## 2. Microfluidic Device Fabrication

Highly parallel microfluidic devices have been fabricated through direct fs laser machining of fused silica followed by chemical etching. Long internal features, such as microfluidic channels for applications in biotechnology, have typically been written with a tightly focused laser spot by translating the substrate so as to overlap a large number of the small volumes affected by individual laser pulses [18]. Similarly, arrays of long, submicron-wide channels have been rapidly formed in glass by reshaping a fs laser beam to a tight line focus and translating the substrate [19]. Fused silica (SiO_2_) was chosen for device substrates because of its high surface quality and low autofluorescence in the red spectral region.

4′ (101.6 mm) SiO_2_ wafers were diced into square chips 14 mm on a side and then annealed at 1140 °C to relieve residual stress and prevent fracturing during laser machining. The annealed chips were cleaned in acetone, rinsed in distilled water, and then mounted on sacrificial borosilicate glass substrates with an adhesive wax. A pneumatic drill with a diamond-coated bit was used to drill access holes through the SiO_2_ chip for sample fluid loading. Drilled chips were then mounted in the fs laser machining setup.

The fs laser machining rig is constructed around a microscope. 200 fs-long laser pulses at 800 nm are produced by a Ti:Sapphire oscillator pumped by a solid-state 532 nm diode laser. The fs beam from the oscillator is then passed through a regenerative amplifier, yielding a maximum average power of 1.2 W at a pulse rate of 250 kHz. The regenerative amplifier may be externally controlled, allowing great flexibility over the pulse rate, even to the point of firing single pulses on demand. The beam's access to the microscope is governed by a shutter, and its power is adjusted with neutral density filters.

Chips to be machined are placed in a chuck attached to motorized stages, located directly beneath the microscope turret, providing nanometer-resolution positioning in three dimensions. A linear gauge mounted in one of the turret's slots may be rotated into contact with the chip surface. The gauge has 0.1 μm resolution and is used both to level the chip and scan the surface region of interest for significant height variations, which may later be compensated for during the machining process. The regenerative amplifier (in external control mode), shutter, stages, and linear gauge are all controllable by the user through a custom LabVIEW program.

Once the chip has been leveled and aligned, fluid access macrochannels are machined from each of the drilled holes toward the central region between the holes using a 32× dry objective and high average power of 1 W at the nominal pulse rate of 250 kHz. The macrochannels are approximately 25 μm deep and start out 100 μm wide at the hole but flare out to 1000 μm wide at the ends. A 500 μm long plateau gap in which the microchannels are to be machined is left between the macrochannels.

Far different conditions must be used to machine the microchannels. Whereas low magnification and high power are employed for the macrochannels, much lower power and higher magnification are necessary for the microchannels. A 160× water-immersion objective with a 1.2 numerical aperture focuses the beam to a submicron beam waist. In addition to reducing the power, the pulse rate and stage velocity must also be configured to ensure that the energy delivered to the focal volume is not too high, in order to avoid damage spreading too far, such as reaching into neighboring channels. Thus, the pulse rate is lowered to 600 Hz, the average power is reduced to 400 μW, and the chip is translated at 300 μm/s. For these parameters, only about two pulses arrive at the laser focus per micron, resulting in well-localized, submicron-scale damaged volumes.

Microchannel lines are written in several layers, starting about 3 μm below the chip surface and translating the focus toward the surface in successive layers. The channels can be made deeper, but their depth should not exceed the depth of focus (∼3 μm) in the experimental microscope, because fluorescently labeled molecules outside the focal region may also be excited and thereby degrade the signal-to-noise ratio (SNR).

Ideally, the laser damage should just barely break the chip surface so that etchant can access the entire microchannel quickly. The power must be reduced even further to 300 μW to achieve this minimal surface damage. Adding a process text identifier label to the chip is the final machining step. The label also aids orientation and positioning. Pulse and translation parameters were determined empirically to yield consistently machined microchannels.

The machined chip is removed from the glass substrate, cleaned of wax in acetone, and rinsed in distilled water. The chip is then etched in a 10 M aqueous solution of potassium hydroxide (KOH) at 80 °C, which results in a 350:1 selective etching of laser-damaged to undamaged fused silica [17]. The process cleans off any large debris scattered across the surface from the macrochannel machining and also clears out the microchannels. The optimal etching time depends on the size and depth of the features and may be experimentally determined. In this work, adequate etching was obtained after one hour. Longer etching times may be used as necessary, since KOH etches undamaged SiO_2_ at a much slower rate of about 7 nm/min [20]. However, etching for too long may lead to undesirable effects, such as erosion of the interchannel separations or enlarged surface craters.

**Figure 1.**
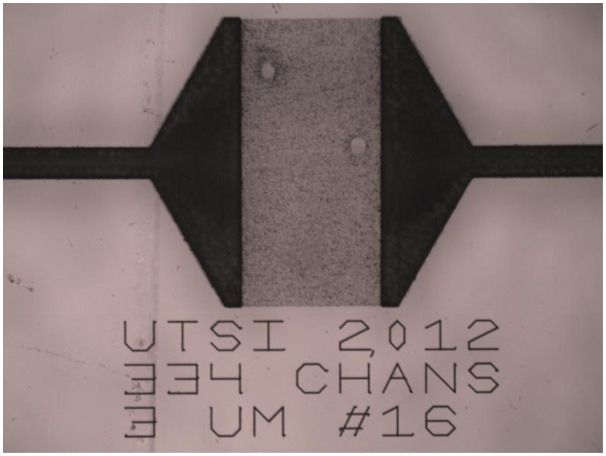
Optical micrograph of microchannels machined in SiO2 and etched for one hour in hot 10 M KOH solution. The large, flared dark areas on the right and left sides are the macrochannels leading to the access wells (not shown).

The etched chip is thoroughly rinsed and sonicated in distilled water. Characterization of machined features may be performed through a variety of methods, including optical microscopy, atomic force microscopy, profilometry, or direct scanning electron microscopy (SEM) of metallized samples [14–16,18]. Figure 1 shows an optical micrograph at 10× magnification of the microchannel region. For scale, the text letters are 100 μm high. The macrochannels leading to the fluid access wells are clearly visible on either side as the dark, flared structures. As the text declares, 334 microchannels with 3 μm pitch were written across the gap.

**Figure 2.**
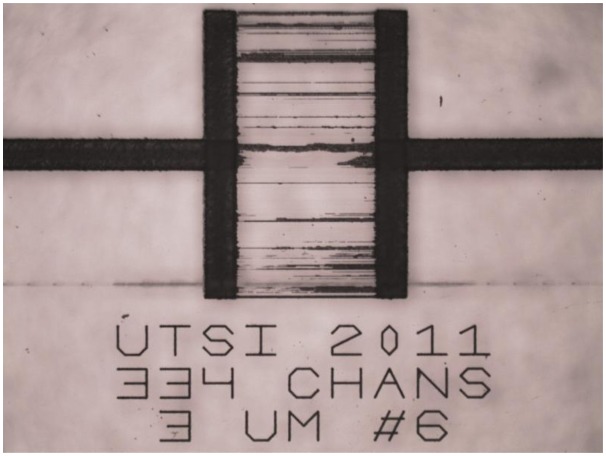
Optical micrograph of microchannels machined in SiO2 and over-etched in hot 10 M KOH solution for 24 h.

Figure 2 shows an example of a previous attempt at machining microchannels where the chip was etched for too long in KOH, in this case 24 h. Although undamaged surface regions were not affected, many of the microchannel separations eroded, leaving large scars of connected channels.

For further characterization of microscale features, combining focused ion beam milling with SEM provides an informative but destructive method for investigating the depth and cross-sectional profile of features [15]. Another established, non-destructive technique applicable to narrow, deep features exposed at the surface is to create a replica mold of the features with a cellulose acetate film, metallize it, and view it with SEM [15]. An advantage over optical microscopy is that the overall channel depth and cross-sectional profile can also be observed by tilting the replica to 45°.

To make a replica, a thin film of cellulose acetate is dissolved into the channels with acetone, carefully peeled off and adhered to an SEM sample peg, and sputtered with gold (or other conductive metal). For shallow features such as these microchannels, the chip could be directly sputtered with metal and viewed in the SEM, but then the metal coating would have to be stripped. Following acetate replication, the chip merely needs to be cleaned in acetone. Acetate replicas are especially suitable for revealing deep features with high aspect ratios [15].

In order to create a usable microfluidic device, channels open at the surface must be sealed. However, reversible mechanisms such as simple van der Waals force between two surfaces or tape adhesives cannot withstand high pressures [21]. A relatively quick and simple method is to bond the machined chip to a cover slip spin-coated with a thin film of poly(dimethylsiloxane) (PDMS) [21]. Both substrates are first plasma-cleaned to activate the surfaces, then simply pressed together and baked at 55 °C for 30 min. The PDMS film molecularly bonds to the SiO_2_ surface, resulting in a transparent, permanent seal. Because background signal is always a concern, the autofluorescence of a neat PDMS film pumped at 660 nm was verified to be about the same as SiO_2_ itself for this wavelength.

## 3. Detecting Fluorescent Molecules in a Microfluidic Device

**Figure 3.**
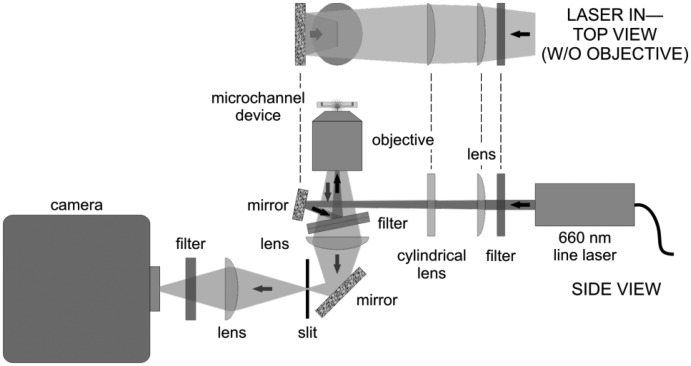
Schematic of the custom wide-field microscope.

A custom wide-field fluorescence microscope, outlined in Figure 3, was built in order to excite and measure fluorescence signals from multiple microchannels simultaneously. The light source for the microscope is a line-generating diode laser that emits 60 mW at 660 nm. The line laser contains an adjustable focusing lens used in conjunction with an additional spherical lens to optimize the thickness of the focused line in the sample. The longer dimension of the beam is focused by a cylindrical lens into an infinity-corrected 60× water-immersion microscope objective with a high numerical aperture of 1.2 and an effective tube lens length of 180 mm. This configuration produces a Köhler-type illumination profile at the focus. The focused line is approximately 5 μm thick and exhibits a near-Gaussian intensity distribution more than 500 μm long, oriented across the channels.

Using a compact epi-illumination format, the laser beam reflects off an angled long-pass filter into the objective. The filter rejects scattered and reflected laser light while passing generated fluorescence. The fluorescence is collected and focused by an f = 50 mm lens through an adjustable slit oriented perpendicular to the microchannels and located at the focal point of the lens. Because of space requirements, a second f = 50 mm lens is needed to refocus the fluorescence onto an EM-CCD camera array without affecting the magnification. The effective system magnification is thus 16.7×, and the EM-CCD array consists of 512 × 512 pixels, where each pixel is 16 μm square. Approximately 1 μm in object space maps to 1 pixel in image space. Hence, a microchannel that is 1 μm wide is imaged onto 1 pixel column. A second long-pass filter is placed before the camera to remove any remaining scatter and background.

**Figure 4.**
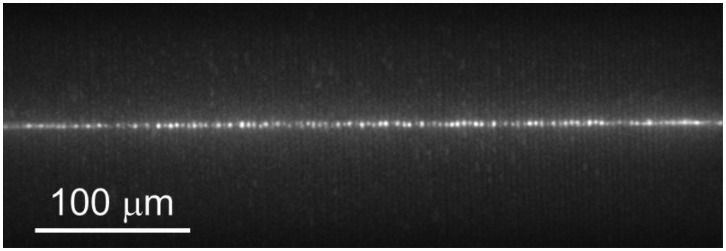
Laser line focused in fluid-filled microchannels. The slit is wide open.

After placing a bonded microchannel device in a custom-built fluidic housing, the housing is mounted in the wide-field microscope and positioned so that microchannels fill the field of view using diffuse white light and with the slit in Figure 3 wide open. A 1 nM aqueous solution of fluorescently labeled protein molecules (streptavidin labeled with Alexa Fluor^®^ 660, Life Technologies, Grand Island, NY, USA) is then loaded into the microchannels with pressure-driven flow initiated by manually actuating a syringe, and the laser line is focused within the depth of the microchannels, as seen in Figure 4. Molecules will be excited and fluoresce as they flow perpendicularly through the laser excitation region within any particular channel.

The slit is closed down once the microchannels are aligned and the laser properly focused. Only the vertical section of the microchannels within the laser focal region, about 5 μm (or 5 pixels) high, is imaged on the EM-CCD. The image is relocated to the bottom of the EM-CCD array by translating the camera vertically. Focusing the fluorescence signal onto only a single row of pixels is unnecessary because the EM-CCD camera can bin adjacent pixels while ignoring the rest of the array. When binned, the charges from each column of the bottom 5 pixel rows are collected to form one “superpixel” row.

The camera also contains a second, identical EM-CCD array that is used as a frame transfer storage buffer. Data collection rates of up to 7.1 kHz may be maintained indefinitely when binning is employed with the frame transfer capability. For low signal levels, an electron-multiplying gain factor of up to 300 may be applied without retarding the collection time. However, a poor SNR will not improve much by applying large gains, as background will also be enhanced. The collection rate may be increased slightly by focusing onto a single row of pixels and eliminating binning.

Data collection proceeds while solution flows through the microchannels. Photoelectrons accumulate in binned pixels for about 130 μs when the collection rate is 7.1 kHz. Once the accumulated charge is transferred to the buffer array, the main array is ready for the next accumulation. As the buffer array fills, each row of data is spooled directly to a file as a single frame of 1 × 512 pixels. Data is thus collected for a specified number of frames, R.

The acquired data file is read into Matlab with an m-file script and reshaped into a single array of R rows × 512 columns. After subtracting background, a matched digital Gaussian filter based on the focal volume and flow velocity is applied. The processing time is relatively fast (depending on the computer's processor and memory) because all operations are array-level, making the digital filtering a quick and simple means of checking data for fluorescence signals. For a Windows 7 desktop running a 3.40 GHz Intel^®^ Core™ i-7 processor and 8 GB of RAM, the m-file script requires less than 2 s to analyze an R = 30,000 frame data file.

A data set was simulated to test the filtering process. Simulated fluorescence photons were generated and detected by the EM-CCD camera according to typical experimental conditions as the photoelectron count rate for a single microchannel [22]. For the simulation, the microchannel had a 1 μm^2^ cross-section, and the laser excitation region was considered to have uniform intensity over this cross-section but followed a Gaussian profile along the microchannel axis, with a 1 μm beam waist and a power of 64 μW. The solution concentration was 1 pM, and the flow velocity was set at 500 μm/s. The fluorescence was assumed to be focused onto a single row on the EM-CCD array, in order to minimize the acquisition time at 122 μs, and the EM gain was set at 300. The simulated background level of 1550 counts/s was determined to be consistent with experiment by illuminating a blank substrate with the laser for these camera settings. Details concerning photon generation photophysics, detection with an EM-CCD, and matched digital filtering are found in References [22–24].

The simulation produced a 1D file of photoelectron count rate data for a single channel. In the actual device, 1 μm-wide microchannels are separated by 2 μm of SiO_2_, so approximately 170 microchannels can be imaged onto the 512 pixel EM-CCD array. To correspond to the experimental microfluidic device and EM-CCD dimensions, the 1D simulation data was therefore reshaped into a 2D array consisting of R = 4820 “frames” and 170 columns containing the photoelectron count rate data (*i.e.*, “microchannels”), each separated by two spacer columns (assigned values of the background count rate plus random fluctuations about this level of up to 10% of the average count rate for the entire data set), thereby creating a 512-column array modeling the experimental device.

**Figure 5.**
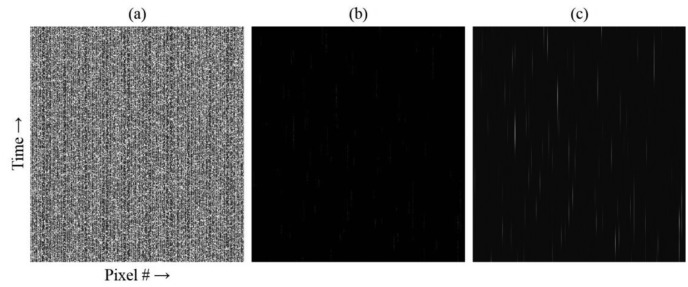
M-file script assessment of simulated fluorescence data. (**a**) The array has been reshaped and the background subtracted; (**b**) the data in (a) has been normalized; (**c**) a matched digital Gaussian filter has been applied to the data in (a) and the result normalized. Only a portion of the total data collection time is shown.

The m-file script assessment of the simulated data is shown in Figure 5. The SNR for this simulation was 164:1. The reshaped raw data minus background for a time segment corresponding to ∼69 ms (570 frames) is shown in Figure 5a. Fluorescent bursts from molecules are not visually apparent above the noise level. For a flow rate of 500 μm/s, a molecule should transit the focus in 2 ms (ignoring diffusion), or over about 16 frames. Because of the photophysics, the photons are not emitted continuously but in bursts [22]. Normalizing to the maximum of the data set in Figure 5a allows these bursts to be viewed in Figure 5b, although the contrast is poor. However, by first applying a matched digital Gaussian filter and then normalizing, streaks representing individual molecules passing through the focus become readily apparent, as seen in Figure 5c. Note that the application of the matched digital filter artificially stretches the elapsed time, so that the streaks appear much longer than the 2 ms transit time.

**Figure 6.**
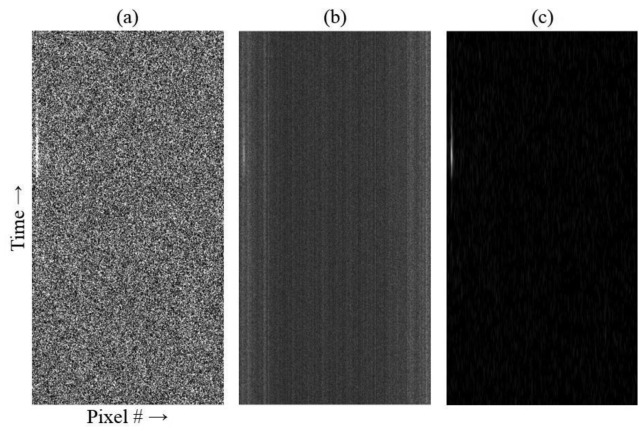
M-file script assessment of experimental fluorescence data. (**a**) The array has been reshaped and the background subtracted; (**b**) the data in (a) has been normalized; (**c**) a matched digital Gaussian filter has been applied to the data in (a) and the result normalized (contrast has been further enhanced to increase visibility). Only a portion of the total data collection time is shown.

The m-file script assessment of the experimental data is shown in Figure 6. Figure 6a depicts the raw data minus background for a 70 ms time segment (500 frames, 139 μs per frame). The background level in the experiment was much higher than in the simulation, resulting in an SNR of only 3:1. For this device, the PDMS film bonded poorly over the microchannel region of the chip because of edge projections bordering the microchannels, which occurred as ejected molten material rapidly re-solidified during the machining process. This imperfect bond thus allowed a shallow, fluid-filled gap to form above the microchannels where fluorescent molecules escaped confinement in the microchannels. Though not in focus, this reservoir contributed to the background.

Normalization to the global maximum of the data set has again been performed on the data shown in Figure 6b. Because the SNR is low, normalization does not provide any new visual confirmation of fluorescence bursts. Applying a matched digital Gaussian filter enhances the bright streak seen on the left of 6a, as well as revealing numerous very faint streaks and a pair of slightly brighter streaks near the lower right in Figure 6c. Note that the contrast in this image has been further enhanced to improve the visibility of the faint streaks. Because the matched digital filtering method relies on computational values, the visual appearance of streaks is convenient but unnecessary, as long as the process can differentiate fluorescence signals above the background.

It can be seen that the brightest streak at left is not confined within a single column, as was the case with the simulated data. This is because the mapping between object and image space is not exactly 1:1, and it is not possible to align microchannels and pixel columns perfectly across the image. Some overlap occurs between adjacent pixel columns, especially farther away from the image center.

More in-depth analysis in the form of an autocorrelation of the raw fluorescence data for each microchannel can be calculated once fluorescent signals have been verified. However, the autocorrelation is computationally much more intense, especially for large data files: the simulated data correlation requires ∼2 min, but the experimental data requires ∼1.5 h (on the same computer as described above), because looped operations must be performed on the individual columns. Thus, the matched digital filtering algorithm is first applied to verify the presence of fluorescence bursts.

**Figure 7.**
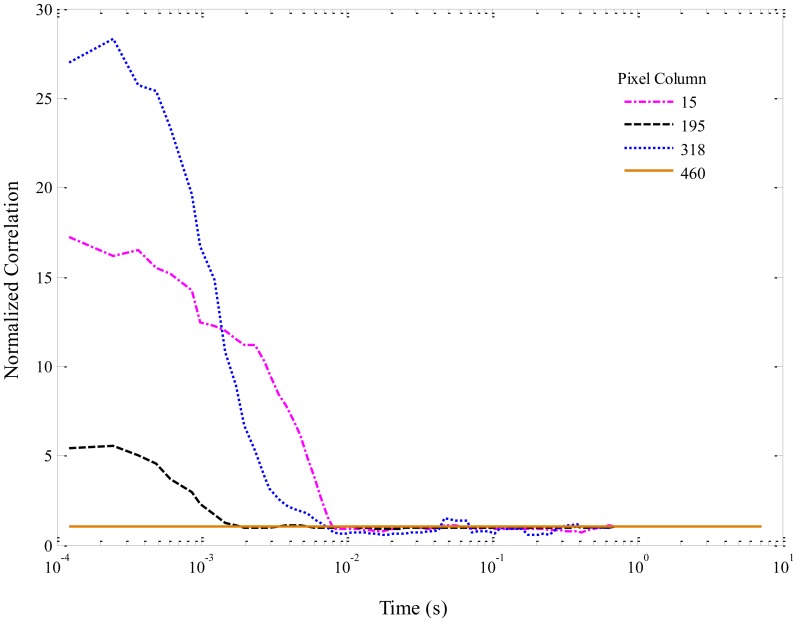
Normalized autocorrelation functions for selected columns of the simulated data in Figure 5.

Normalized autocorrelation functions for selected columns of the simulated data in Figure 5 are shown in Figure 7. The curves have been smoothed with a 5-point moving average. For viewing clarity, only a few curves are plotted. Columns were chosen based on the maximum and minimum amplitudes of the autocorrelations, in order to be representative of the overall correlation amplitudes. In Figure 7, column 318 contains the maximum overall correlation amplitude, while column 460 corresponds to a spacer and thus shows no correlation. The other two columns lie between these extremes. The autocorrelation amplitudes, being inversely related to the number of molecules in the focal region, are high for the simulated data because well-separated molecules in a very dilute solution were modeled. It is interesting to note that each column also demonstrates a slightly different flow velocity, which is directly related to the width of the correlation peak (*i.e.*, the time a molecule spends passing through the excitation region), because of the effects of diffusion that were included in the simulation.

**Figure 8.**
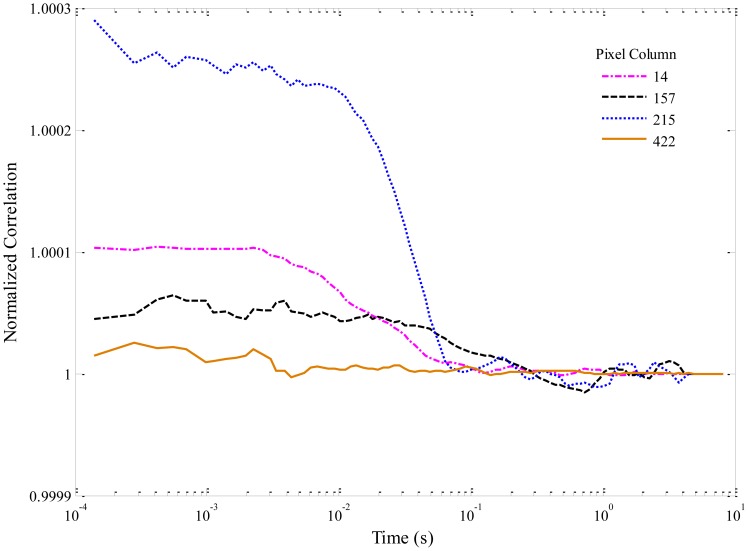
Normalized autocorrelation functions for selected columns of experimental data in Figure 6.

Selected autocorrelations for the experimental data in Figure 6 are shown in Figure 8. The curves have again been smoothed. Columns were again chosen following the same reasoning.

The maximum correlation occurs not for column 15 (with the brightest streak in Figure 6c) but for column 215, corresponding to the pair of fainter streaks noted earlier. Two main differences from the simulated data can be observed. First, the amplitudes of the correlations are very small, because of both the lower SNR and the higher concentration used (1 nM). Second, the widths of the correlation peaks (∼20 ms) are about an order of magnitude higher than in the simulated case. The average flow velocity extrapolated from the correlations is thus about 50 μm/s, as compared to 500 μm/s in the simulation.

Assuming an individual microchannel's cross-section to be 1 μm × 3 μm and if the laser focus extends 5 microns along the microchannel, the excitation volume is 15 μm^3^ (15 fL). The Alexa Fluor^®^ 660 streptavidin conjugate has 4:1 loading (*i.e.*, 4 dye molecules per streptavidin molecule), so for 1 nM concentration, the solution should contain about 6/4 × 10^14^ molecules/L, or 0.15 molecules/fL. Thus, the static excitation volume per channel should contain roughly two streptavidin molecules on average. For a flow velocity of 50 μm/s, about 20 molecules should then pass through the excitation region per second. The number of streaks per column observed in Figure 6c is commensurate with this estimation.

## 4. Conclusions

We have demonstrated a high-throughput microfluidic system with sensitive, fluorescence-based detection by an EM-CCD array. A highly parallel microchannel device was constructed by fs laser machining of fused silica and subsequent wet etching. The device was tested in a custom-built, wide-field microscope with epi-illumination from a line-generating laser operating in the deep red. A dilute aqueous solution of fluorescently labeled proteins was loaded into the microchannels and photoelectron count data were collected at a rate of 7.1 kHz. The presence of fluorescence signals was initially confirmed through matched digital filtering. Fluorescence autocorrelations were performed to provide further analysis. A simulated set of fluorescence data was generated and analyzed with the same procedures for comparison.

Although this work is unique in its ability to detect fluorescence responses from single, fluorescently labeled biomolecules in 170 fluidic channels simultaneously, the devices described in this paper are prototypes in development, and various aspects of the fabrication and experimental processes remain to be optimized: optical performance of the microscope, flow velocity calibration with a syringe pump, minimum acceptable concentration, microchannel uniformity and quality, increasing the density of microchannels to the target of 196, *etc.* Fused silica substrates are relatively costly, and the overlap-scanning fs laser machining process described here is poorly suited to rapid mass-production of microfluidic devices. However, imprint lithography of affordable polymer wafers may still offer an avenue to increasing microchannel production capability, despite past difficulties in lithography of micron-scale channels [13]. Alternatively, single-shot line-focused laser machining may offer another means of rapidly producing an array of parallel microchannels [19].

Following optimization, highly parallel microfluidic devices would be applied to screening therapeutic candidates against specific targets, such as the enzyme L1 endonuclease. This enzyme has been shown to cause double-strand breakage in DNA and is linked to aging and numerous diseases, including cancer [25]. Screening a small library of potential drug candidates with L1 endonuclease would provide a straightforward evaluation of the device's usefulness for therapeutic drug assays.

## References

[1] Sundberg S.A. (2000). High-throughput and ultra-high-throughput screening: Solution- and cell-based approaches. Curr. Opin. Biotech..

[2] Olsen M., Iverson B., Georgiou G. (2000). High-throughput screening of enzyme libraries. Curr. Opin. Biotech..

[3] Li Y., Buch J.S., Rosenberger F., DeVoe D.L., Lee C.S. (2004). Integration of Isoelectric Focusing with Parallel Sodium Dodecyl Sulfate Gel Electrophoresis for Multidimensional Protein Separations in a Plastic Microfluidic [*sic*] Network. Anal. Chem..

[4] Yu M., Wang Q., Patterson J.E., Woolley A.T. (2011). Multilayer Polymer Microchip Capillary Array Electrophoresis Devices with Integrated On-Chip Labeling for High-Throughput Protein Analysis. Anal. Chem..

[5] Gerlach A., Knebel G., Guber A.E., Heckele M., Herrmann D., Muslija A., Schaller T. (2002). Microfabrication of single-use plastic microfluidic devices for high-throughput screening and DNA analysis. Microsyst. Technol..

[6] Mckenna B.K., Selim A.A., Bringhurst F.R., Ehrlich D.J. (2009). 384-Channel parallel microfluidic cytometer for rare-cell screening. Lab Chip.

[7] Schonbrun E., Abate A.R., Steinvurzel P.E., Weitzab D.A., Croziera K.B. (2010). High-throughput fluorescence detection using an integrated zone-plate array. Lab Chip.

[8] Hatch A.C., Fisher J.S., Tovar A.R., Hsieh A.T., Lin R., Pentoney S.L., Yang D.L., Lee A.P. (2011). 1-Million droplet array with wide-field fluorescence imaging for digital PCR. Lab Chip.

[9] Colyer R.A., Scalia G., Villa F.A., Guerrieri F., Tisa S., Zappa F., Cova S., Weiss S., Michalet X. (2011). Ultra high-throughput single molecule spectroscopy with a 1024 pixel SPAD. Proc. SPIE.

[10] Sivanesan P., Okamoto K., English D., Lee C.S., DeVoe D.L. (2005). Polymer Nanochannels Fabricated by Thermomechanical Deformation for Single-Molecule Analysis. Anal. Chem..

[11] Xu B.-Y., Xu J.-J., Xia X.-H., Chen H.-Y. (2010). Large scale lithography-free nano channel array on polystyrene. Lab Chip.

[12] Liu J., Qiao H., Xu Z., Liu C., Wang J., Du L., Zhang X., Wang L. (2012). Fabrication of planar nanofluidic channels in thermoplastic polymers by O_2_ plasma etching. Micro Nano Lett..

[13] Okagbare P.I., Soper S.A. (2010). Polymer-based dense fluidic networks for high throughput screening with ultrasensitive fluorescence detection. Electrophoresis.

[14] Liu X., Du D., Mourou G. (1997). Laser ablation and micromachining with ultrashort laser pulses. IEEE J. Quantum Electron..

[15] White Y.V., Li X., Sikorski Z., Davis L.M., Hofmeister W. (2008). Single-pulse ultrafast-laser machining of high aspect nano-holes at the surface of SiO_2_. Opt. Expr..

[16] Zalloum O.H.Y., Parrish M., Terekhov A., Hofmeister W. (2010). On femtosecond micromachining of HPHT single-crystal diamond with direct laser writing using tight focusing. Opt. Expr..

[17] Kiyama S., Matsuo S., Hashimoto S., Morihira Y. (2009). Examination of Etching Agent and Etching Mechanism on Femotosecond Laser Microfabrication of Channels inside Vitreous Silica Substrates. J. Phys. Chem. C.

[18] Farson D.F., Choi H.W., Zimmerman B., Steach J.K., Chalmers J.J., Olesik S.V., Lee L.J. (2008). Femtosecond laser micromachining of dielectric materials for biomedical applications. J. Micromech. Microeng..

[19] Davis L.M., Bradfield J.W., Rohde C.A., Simpson M.C. Machining of High-Aspect Micro/Nano-Channels with a Single Femtosecond Laser Pulse Focused to a Line.

[20] Williams K.R., Gupta K., Wasilik M. (2003). Etch Rates for Micromachining Processing—Part II. J. Microelectromech. Syst..

[21] McDonald J.C., Whitesides G.M. (2002). Poly(dimethylsiloxane) as a Material for Fabricating Microfluidic Devices. Acc. Chem. Res..

[22] Robinson W.N. (2011). Monte Carlo Simulations of Single-Molecule Fluorescence Detection Experiments. Ph.D. Dissertation.

[23] Basden A.G., Haniff C.A., Mackay C.D. (2003). Photon counting strategies with low-light-level CCDs. Mon. Not. R. Astron. Soc..

[24] Bunfield D. (1997). Simulation of a Single-Molecule Detection Experiment. M.Sc. Thesis.

[25] Gasior S.L., Wakeman T.P., Xu B., Deininger P.L. (2006). The human LINE-1 retrotransposon creates DNA double-strand breaks. J. Mol. Biol..

